# Supplementary material for: IFITM1 and IFITM3 Proteins Inhibit the Infectivity of Progeny HIV-1 without Disrupting Envelope Glycoprotein Clusters
Source: Viruses. 2023 Dec 7;15(12):2390. doi: 10.3390/v15122390 (PMC10748374; doi:10.3390/v15122390)
Supplement: Supplementary file 1 [file viruses-15-02390-s001.zip › viruses-2625497-supplementary.pdf]

IFITM1 and IFITM3 Proteins Inhibit Infectivity of Progeny HIV-1 without Disrupting Env Clusters

Smita Verma[1], Yen-Cheng Chen[1,§], Mariana Marin[1,2], Scott E. Gillespie[1] and Gregory B. Melikyan[1,2,*]

[1] Department of Pediatrics, Emory University School of Medicine, Atlanta, Georgia, USA

[2] Children's Hospital of Atlanta, Atlanta, Georgia, USA

Supplemental Figure Legends

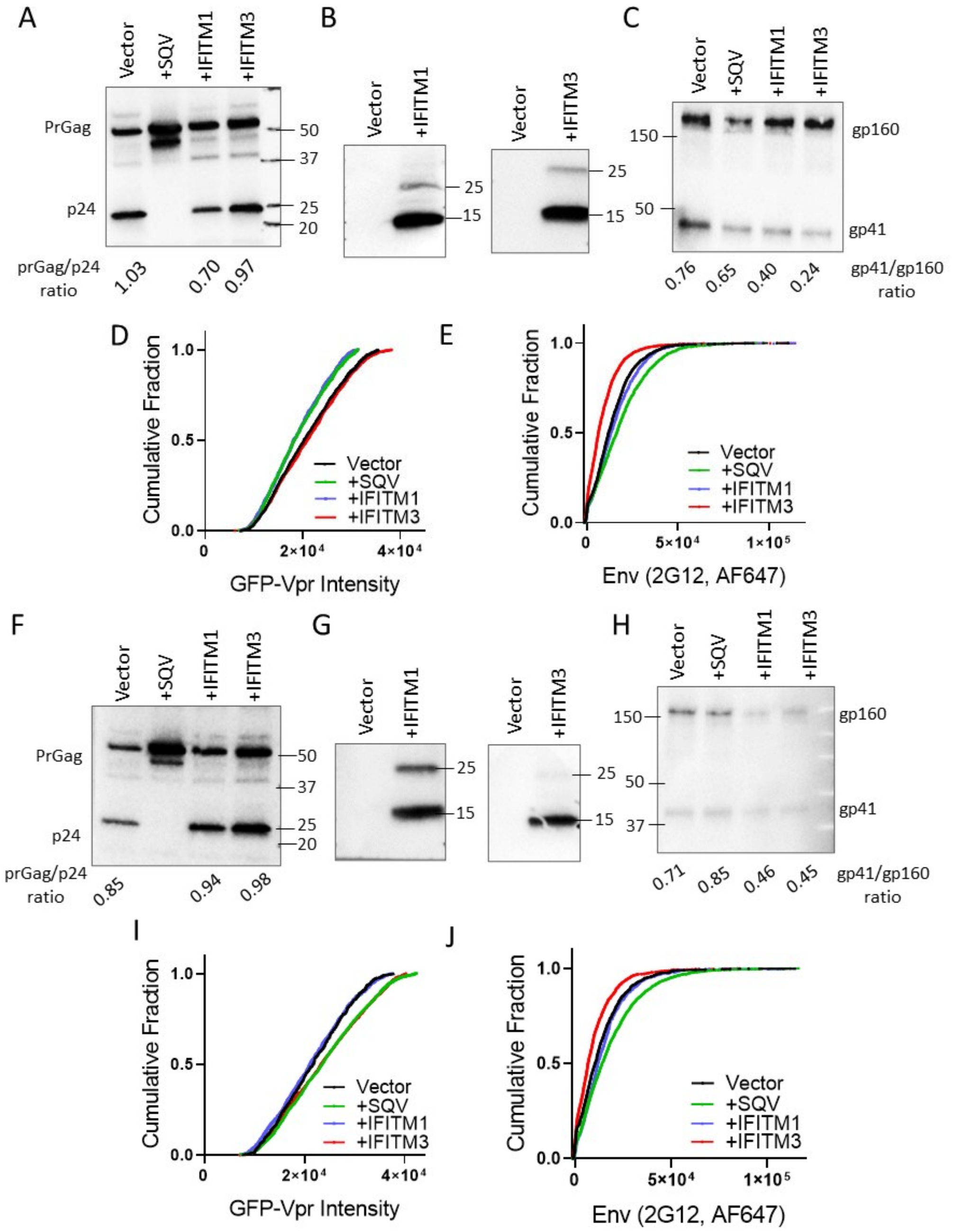

Figure S1. Immunoblotting and immunofluorescence analysis of two independent HXB2 HIV-1 pseudovirus panels. Two pseudovirus panels, each consisting of four preparations: Control (Vector), +SQV (saquinavir treated, immature), IFITM1, and IFITM3 viruses were produced by transfection of HEK293T/17 cells, as described in Methods. (A, F) Characterization of pseudovirus maturation by Western blotting for p24. (B, G) Assessment of IFITM incorporation into pseudoviruses by Western blotting. (C, H) Analysis of HIV-1 Env incorporation and cleavage. Immunofluorescence analysis of GFP-Vpr incorporation (D, I) and HIV-1 Env incorporations (E, J) into single virions using anti-gp120 2G12 primary antibody and anti-human AF647-conjugated secondary antibody.

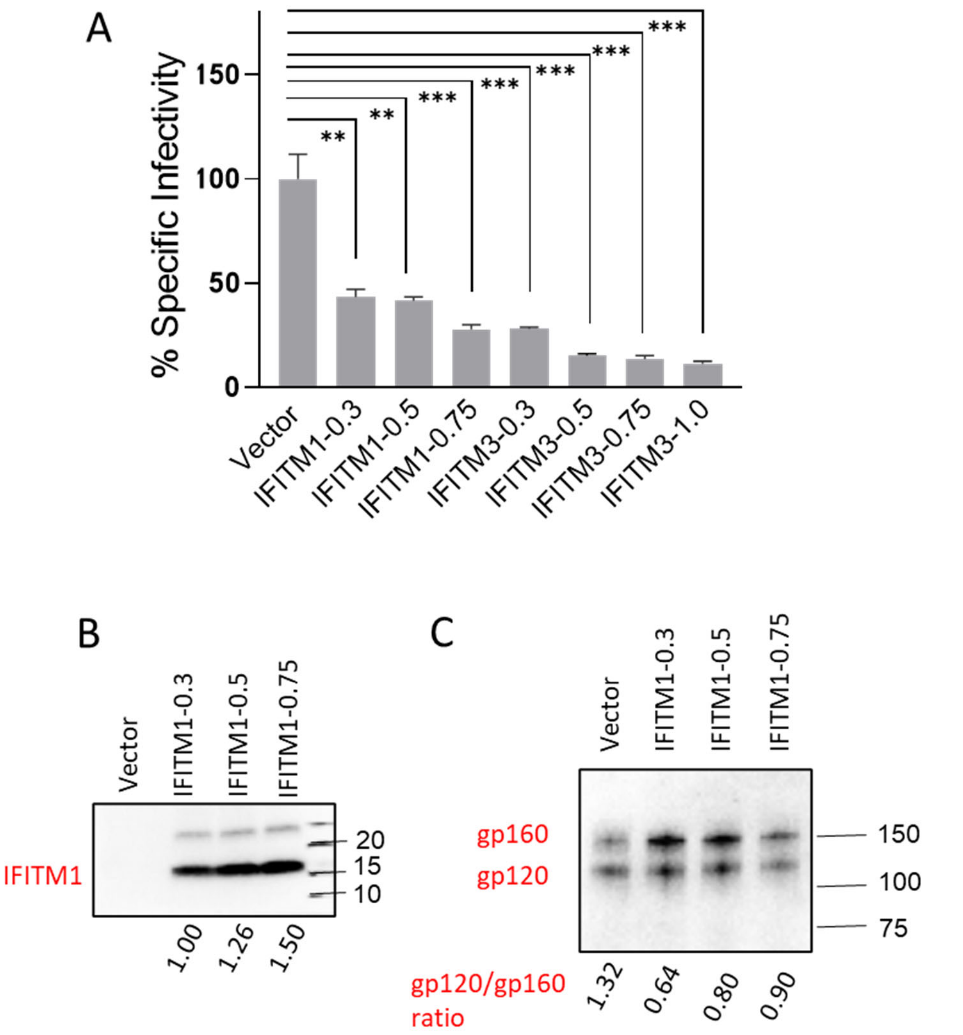

Figure S2. Titration of HIV-1 HXB2 pseudoviruses with increasing amounts of IFITM plasmids. (A) A panel of three pseudoviruses – Vector (Control), IFITM1, and IFITM3 containing particles – was produced in parallel by transfection of HEK293T/17 cells. (A) Dependence of HIV-1 pseudovirus infectivity on the amount of IFITM plasmids used for transfection. TZM-bl cells were infected for 48 h with the indicated pseudovirions using the same amount of viral p24. The resulting luciferase signal is normalized to control (Vector) particles. The statistical analysis was performed using Student's t-test. Significance: n.s., p>0.05; **, 0.01>p>0.001; ***, p<0.001. (B) Western blotting analysis of IFITM1 incorporation into virions upon increasing the plasmid concentration from 0.3-0.75 μg. (C) Immunoblot analysis of Env incorporation and processing for a representative pseudovirus panel produced using increasing amounts of IFITM plasmids.

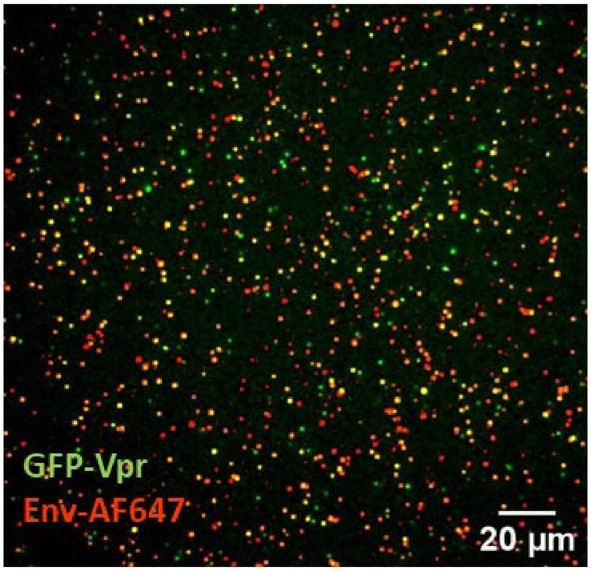

Figure S3. Representative immunofluorescence image of HXB2 pseudoviruses labeled with GFP-Vpr and immunostained for Env. Images of coverslip-immobilized single pseudoviruses show the extent of colocalization of GFP-Vpr (green) with HXB2 Env glycoproteins (red) after immunostaining for Env glycoproteins using anti-gp120 2G12 antibody and AF647-conjugated secondary antibody.

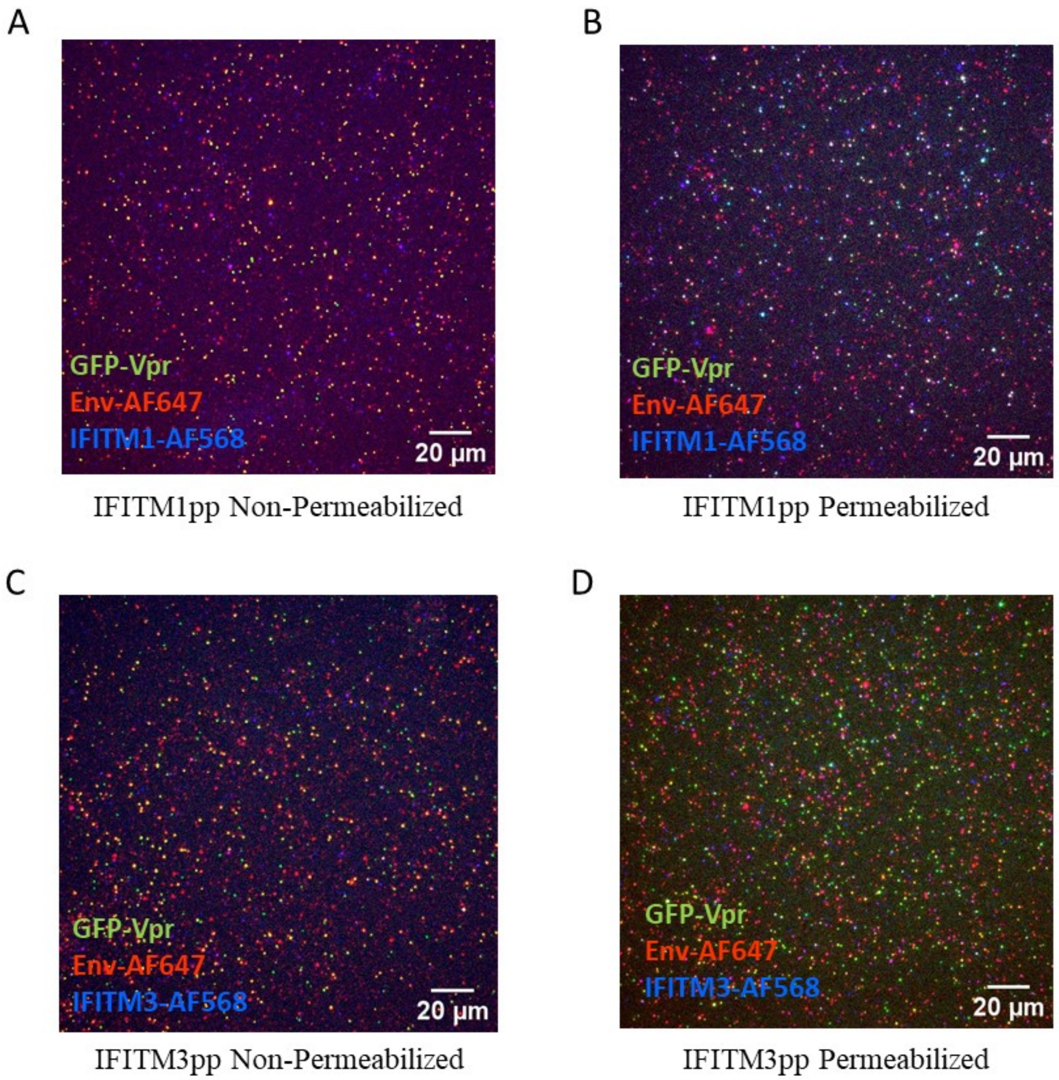

Figure S4. Single virus-based immunofluorescence analysis of IFITM and HXB2 Env incorporation. The virions were fixed and permeabilized using 0.2% Triton-X100. Single virus colocalized image of GFP-Vpr (green) labeled pseudovirions stained for Env glycoproteins (red) and IFITM (blue) proteins.

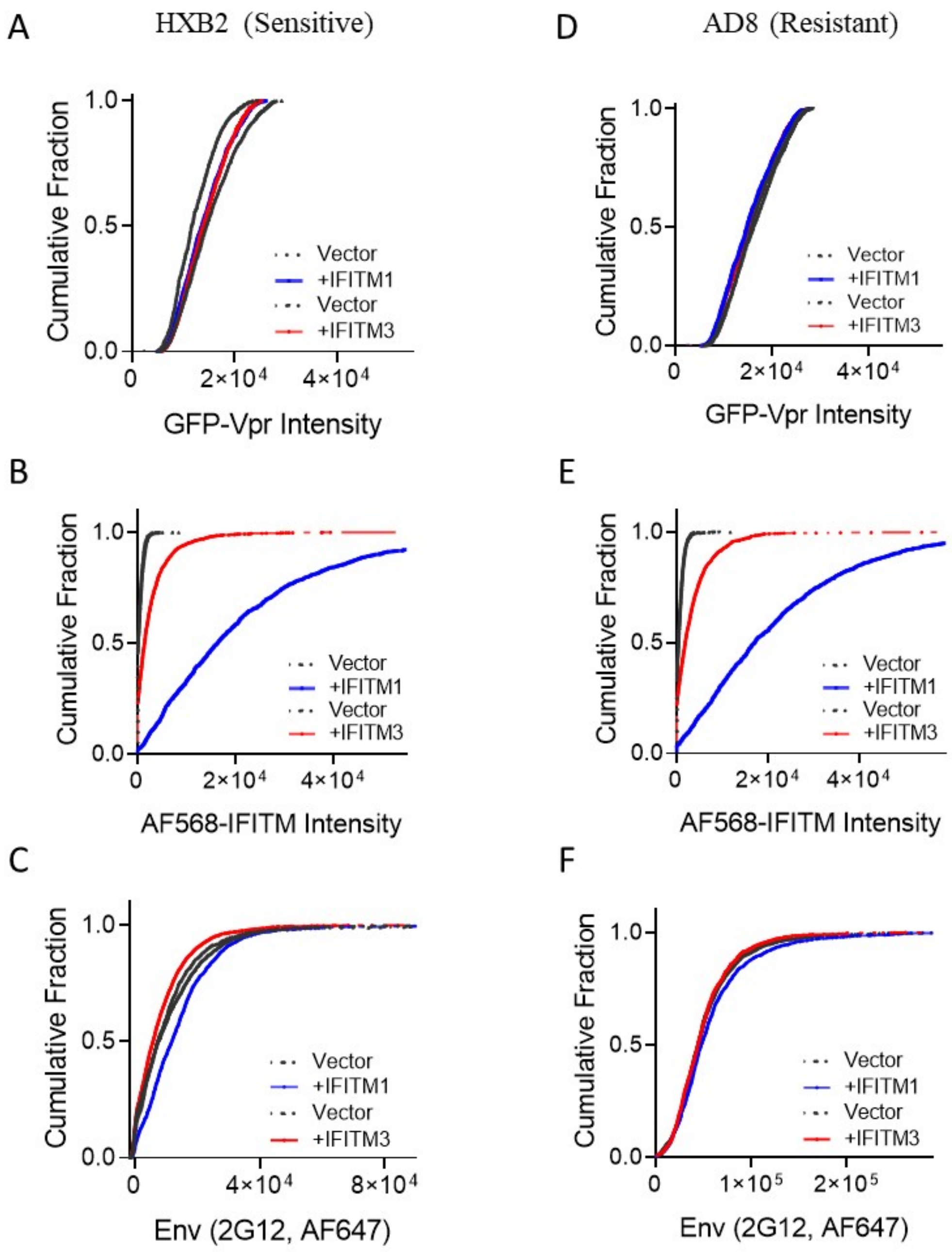

Figure S5. Assessment of IFITM and Env incorporation into single pseudoviruses. Immunofluorescence analysis of HIV-1 GFP-Vpr (A, D), IFITM (B, E), and Env (C, F) fluorescence intensity distributions using single pseudoviruses bearing sensitive (HXB2) and resistant (AD8) Env after fixation and permeabilization with 0.2% Triton-X100.

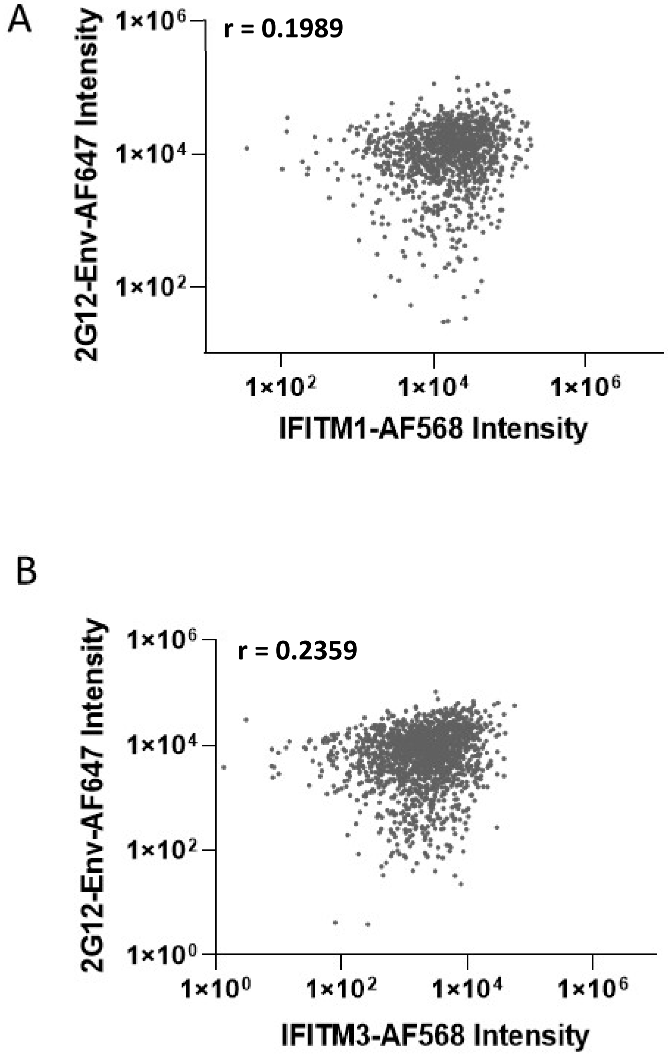

Figure S6. Incorporation of sensitive Env into pseudoviruses does not correlate with IFITM incorporation. (A) Lack of correlation between Env and IFITM1 signals per single virions. (B) Lack of correlation between Env and IFITM3 signals per single virion. Correlation coefficients were calculated using a Pearson correlation coefficient in GraphPad Prism.

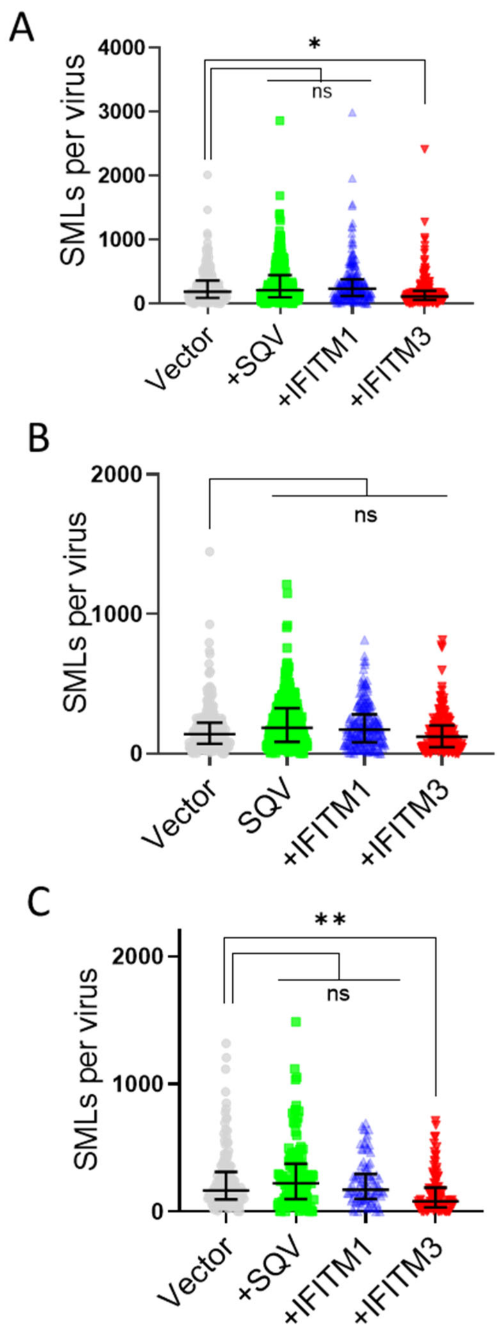

Figure S7. Single-molecule localization analysis of Env incorporation into pseudovirions after optimal binning of SML data. (A-C) Distributions of single-molecule localizations (SMLs) per virion measured by 2D dSTORM for three independent pseudovirus panels. Statistical analysis was performed by a two-sample Kolmogorov−Smirnov (KS) test with optimal binning of data using a custom MATLAB script. Significance: n.s., $p>0.05$; *, $0.05>p>0.01$; **, $0.01>p>0.001$.

A

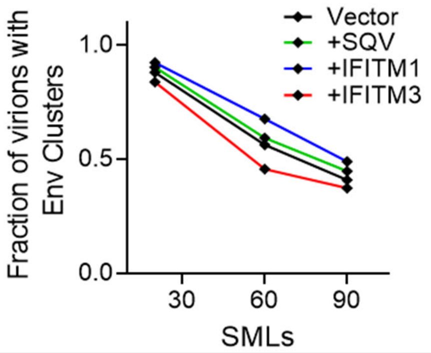

FE test	≥20SMLs	≥60SMLs	≥90SMLs
Control vs SQV	n.s.	n.s.	n.s.
Control vs IFITM1	n.s.	**	n.s.
Control vs IFITM3	**	***	**
IFITM1 vs IFITM3	***	***	***

B

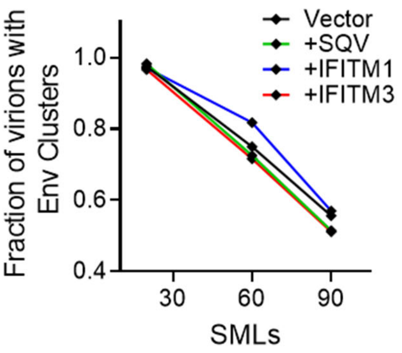

FE test	≥20SMLs	≥60SMLs	≥90SMLs
Control vs SQV	n.s.	n.s.	n.s.
Control vs IFITM1	n.s.	n.s.	n.s.
Control vs IFITM3	n.s.	n.s.	n.s.
IFITM1 vs IFITM3	n.s.	n.s.	n.s.

C

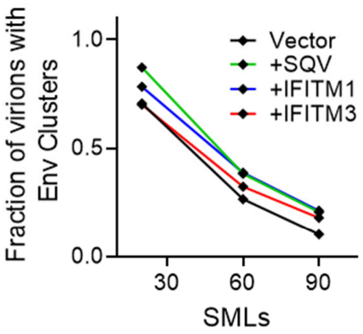

FE test	≥20SMLs	≥60SMLs	≥90SMLs
Control vs SQV	***	***	***
Control vs IFITM1	n.s.	***	***
Control vs IFITM3	n.s.	n.s.	*
IFITM1 vs IFITM3	n.s.	n.s.	n.s.

Figure S8. The effect of IFITMs on Env clustering on HIV-1 pseudoviruses imaged by dSTORM using 2-category analysis. Env clusters were defined by the DBSCAN algorithm using a fixed search radius of 15 nm and varied minimum number of SMLs from ≥20 to ≥90. (A, B, and C) Fractions of pseudoviruses with and without Env clusters as a function of DBSCAN single molecule localization threshold for three independent panels of pseudoviruses. Statistical significance was determined by the Fisher's Exact test. Significance: n.s., $p>0.05$; *, $0.05>p>0.01$; **, $0.01>p>0.001$; ***, $p<0.001$.

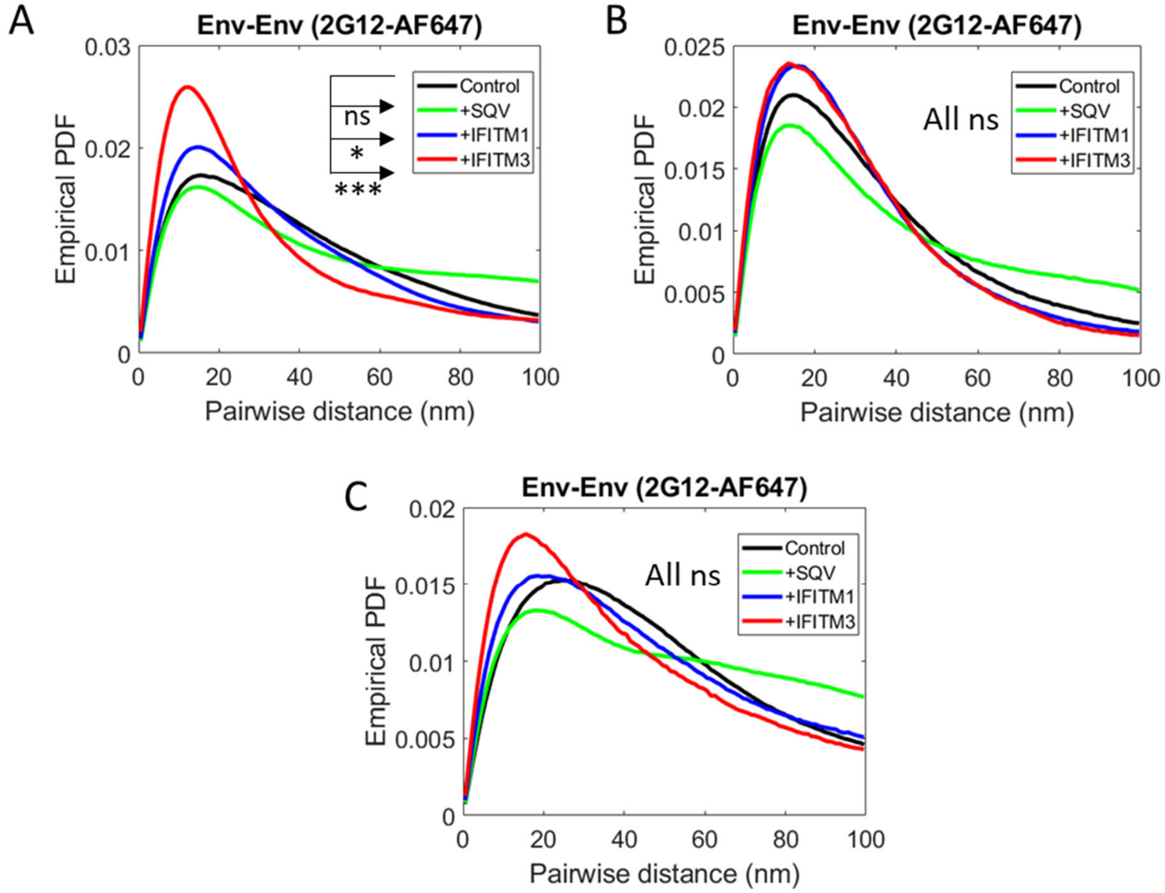

Figure S9. Env-Env pairwise distance distribution analysis on single pseudoviruses. Comparison of the probability density function (PDF) of Env-Env pairwise distances between AF647 SMLs in control (gray), SQV (green), IFITM1 (blue), and IFITM3 (red) pseudoviruses obtained by dSTORM. Statistical analysis was performed by a two-sample Kolmogorov−Smirnov (KS) test with optimal binning of data using a custom MATLAB script. Significance: n.s., p>0.05; *, 0.05>p>0.01; ***, p<0.001.
